# Identification and Validation of Stage-Associated PBMC Biomarkers in Breast Cancer Using MS-Based Proteomics

**DOI:** 10.3389/fonc.2020.01101

**Published:** 2020-07-24

**Authors:** Raheleh Moradpoor, Ahmad Gharebaghian, Farhad Shahi, Asadollah Mousavi, Sina Salari, Mohammad Esmaeil Akbari, Soheila Ajdari, Mona Salimi

**Affiliations:** ^1^Department of Basic Sciences, School of Allied Medical Sciences, Shahid Beheshti University of Medical Sciences, Tehran, Iran; ^2^Laboratory Hematology and Blood Bank Department, School of Allied Medical Sciences, Shahid Beheshti University of Medical Sciences, Tehran, Iran; ^3^Breast Disease Research Center, Tehran University of Medical Science, Tehran, Iran; ^4^Hematology, Oncology and Stem Cell Transplantation Research Center, Tehran University of Medical Sciences, Tehran, Iran; ^5^Medical Oncology, Hematology and Bone Marrow Transplantation, Taleghani Hospital, Shahid Beheshti University of Medical Sciences, Tehran, Iran; ^6^Cancer Research Center, Shahid Beheshti University of Medical Sciences, Tehran, Iran; ^7^Department of Immunology, Pasteur Institute of Iran, Tehran, Iran; ^8^Department of Physiology and Pharmacology, Pasteur Institute of Iran, Tehran, Iran

**Keywords:** PBMC, breast cancer, SWATH mass spectrometry, proteome, secretome, metastasis

## Abstract

**Background:** It is well-described that the transcriptome of peripheral blood mononuclear cells (PBMCs) can be altered in the context of many malignancies to allow them avoid the effective immune response, which leads to cancer invasiveness. Here, we used an MS-based strategy to discover biomarkers in the PBMCs of breast cancer (BC) patients and validated them at different stages of BC.

**Methods:** PBMCs were isolated from the breast cancer patients and were cultured alone or co-cultured with breast cancer cell lines. The role of PBMC in the invasion property of breast cancer cells was explored. NF-kB activity was also measured in the co-cultured breast cancer cells. Identification of protein profiles in the secretome and proteome of the co-cultured PBMCs was performed using SWATH mass spectrometry. Pathway enrichment and gene ontology analyses were carried out to look for the molecular pathways correlated with the protein expression profile of PBMCs in the breast cancer patients. Quantitative real-time polymerase chain reaction (qPCR) was performed to validate the candidate genes in the PBMC fraction of the breast cancer patients at the primary and metastatic stages. *In silico* survival analysis was performed to assess the potential clinical biomarkers in these PBMC subtypes.

**Results:** PBMCs could significantly increase the invasion property of the BC cells concomitant with a decrease in E-cadherin and an increase in both Vimentin and N-cadherin expression. The NF-kB activity in the BC cells significantly increased following co-culturing implying the role of PBMCs in EMT induction. Enrichment analysis showed that the differentially expressed proteins in PBMCs are mainly associated with IL-17, PI3K-Akt, and HIF-1 signaling pathway, in which a set of seven proteins including TMSB4X, HSPA4, S100A9, SRSF6, THBS1, CUL4A, and CANX were frequently expressed. Finally, *in silico* analysis confirmed that a gene set consisting of S100A9, SRSF6, THBS1, CUL4A, and CANX were found to provide an insight for the identification of metastasis in breast cancer patients.

**Conclusion:** In conclusion, our study revealed that the protein expression profile in PBMCs is a reflection of the proteins expressed in the BC tissue itself; however, the abundance level is different due to the stage of cancer.

## Introduction

Tumors that originate from the epithelial cells grow in a dynamic and intricate stroma composed of endothelial cells, blood vessels, immune cells, and a variety of associated tissue-type cells along with matrix proteins and soluble factors (1–3). Indeed, all the required stimuli for tumor growth and progression are directly or indirectly provided by the tumor microenvironment. For example, immune effector cells, which are recruited to the tumor site, have been found to promote tumor growth in response to tumor-derived signals (4–7). In other words, a continual crosstalk between tumor cells and the surrounding stroma including the immune cells leads to development and metastasis of the tumor. It is noteworthy mentioning that metastasis is the main determinant of the clinical outcome of cancer patients (2, 8, 9). Consistently, both clinical and experimental evidence demonstrate the importance of tumor microenvironment in breast cancer invasiveness. Two of the most abundant immune cell populations found in the stroma of breast cancer are tumor-associated macrophages (TAMs) representing up to 50% of the tumor mass and T cells (10–14). Both of them might have been associated with a poor prognosis and detrimental clinical outcome in breast cancer patients, likely by promoting the epithelial to mesenchymal transition (EMT) process, metastasis, and angiogenesis. During EMT, tumor cells exhibit dynamic changes in epithelial and mesenchymal compositions, which endow tumor cells with enhanced invasive and resistance phenotypes (11, 15–17).

Peripheral blood mononuclear cells (PBMCs) as a source of myeloids and lymphoids represent a key feature in the host immune defense system. Upon recruitment of PBMCs to tumor site by tumor cells, they are then converted to tumor promoting immune cells including myeloid-derived suppressor cells (MDSCs) and TAMs by the action of tumor microenvironment factors. This is then followed by the activation of the signaling pathways, including Akt, STAT, and WNK, accompanied by changes in gene expression profiles. MDSCs and TAMs can promote or suppress the host immune response against cancers, including breast cancer by releasing a plethora of cytokines (18–24). TAMs inhibit antitumor immune responses mediated by T cells, and stimulate neoangiogenesis, extracellular matrix remodeling, and the subsequent tumor progression (25, 26). In addition, MDSCs can exert their immunosuppressive function through the induction of CD4+CD25+Foxp3+ regulatory T cells. As cytokines such as CSF-1 and VEGF and chemokines including CCL2 and CCL5 are important mediators in the recruitment and functional polarization of immune suppresser cells in a tumor microenvironment (26), research on the molecular alterations in PBMCs may provide new insights into cancer status.

Several studies have speculated that altered composition of the immune cells is linked to cancer metastasis (10, 27–29). On the other hand, preclinical and clinical studies have shown that EMT and invasion are vital prerequisites for tumor metastasis (1). Based on these observations and the fact that immune cells are an integral component of PBMCs, the objective of the current study is to identify the PBMCs-associated biomarkers correlating with immune-mediated EMT alterations that may be paramount in breast cancer progression. Thus, we established an *in vitro* co-culturing system using the premalignant epithelial breast cancer cell line MCF-7 and PBMCs, freshly isolated from breast cancer patients. We then analyzed the impact of PBMCs on the breast cancer cells in terms of phenotypic changes including the expression of EMT markers, invasion capacity, and NF-κB activity.

Herein, we report that PBMCs, isolated from breast cancer patients, induce invasiveness of breast cancer cells, while those isolated from healthy individuals lack such property. The proteome and secretome profiles of the PBMCs were also analyzed using LC-MS/MS spectrometry after co-culturing with breast cancer cells. Interestingly, these analyses revealed a similar series of biomarkers previously reported in metastatic breast tumors. These biomarkers were further validated in blood samples from patients in the primary and metastatic stages of breast cancer. Finally, a gene set consisting of S100A9, SRSF6, THBS1, CUL4A, and CANX were introduced as potential candidate genes helpful in distinguishing metastatic from non-metastatic breast cancer patients.

## Materials and Methods

### Cell Culture

MDA-MB-231 and MCF-7 breast cancer cells were cultured according to standard protocols. MDA-MB-231, MCF-7, and PBMCs were cultured in a DMEM and an RPMI medium (Gibco-BRL, Rockville, IN), respectively, supplemented with 10% (v/v) fetal bovine serum (FBS), penicillin/ streptomycin (100 IU/mL and 100 μg/mL) (Gibco-BRL, Rockville, IN), and L-glutamine and then maintained at 37°C in a humidified 5% CO_2_ incubator. Once grown to 80% of confluence, MCF-7 or MDA-MB-231 cells were collected and co-cultured with PBMCs. In some experiments, cells were washed with PBS, and fresh serum-free media were added to obtain condition media (CM) after 24 h. Finally, CM was filtered through a 0.22-μm filter.

### Patients and Peripheral Blood Mononuclear Cell (PBMC) Samples

A total of 24 venous blood samples were collected, among which 21 samples were from the patients and 3 from the healthy volunteers. Healthy PBMCs were obtained from the donors who experienced no relevant previous medical history, whereas 21 samples were obtained from the patients whom breast cancer was histologically confirmed before starting therapy (surgery, radiotherapy, or oncology). Additionally, PBMCs of 20 metastatic breast cancer patients were isolated to verify our findings. All samples were collected with informed consent from patients, and the current study protocol was established by the Human Ethics Research Committee of Shahid Beheshti University of Iran.

### Peripheral Blood Mononuclear Cell (PBMC) Isolation and Co-culturing With Breast Cancer Cells

PBMCs were isolated from the buffy coat using a Lymphoprep gradient medium (density, 1.077 g/ml). Freshly isolated PBMCs from each subject were seeded into the upper and breast cancer cells into the lower compartments of Transwell-6 well culture plates (4-μm pore size, SPL Life Sciences, Korea), for performing the invasion and immunoblotting assays. To analyze the proteome and the secretome, PBMCs were cultured into the lower side of 100-mm cell culture dishes and MCF-7 cells into the upper side of the chamber. The ratios of PBMCs and breast cancer cells for co-cultures were established at 7:1 for 5 days.

### Determination and Characterization of EMT Phenotypes

Western immunoblotting was carried out on the co-cultured MCF-7 and MDA-MB-231 cells lines, using antibodies against epithelial (E-cadherin) and mesenchymal (N-cadherin and vimentin) markers. Briefly, co-cultured cells were washed three times with ice-cold phosphate buffered saline (PBS), and then total protein lysis was performed using a lysis buffer [62.5 mM Tris-HCl, pH 6.8, 0.1% sodium dodecyl sulfate (SDS), 50 mM DTT, 25% Glycerol] with protease inhibitors and harvested by scraping and then denatured by boiling at 95°C. Total cell lysates with equal amounts of protein samples per well were separated by 10% SDS-PAGE gel electrophoresis and electro-transferred onto PVDF membranes (GE Health Care Life Sciences, Buckinghamshire, UK) for 2 h at 300 V. The blots were then blocked with 1% w/v casein in Tris-buffered saline (TBS, pH 7.8) for 2 h and probed with primary antibodies overnight at 4°C. The following primary antibodies were used: E-cadherin (1:200), N-cadherin (1:500), vimentin (1:500), and GAPDH (1:1,000). Immunodetection of proteins was performed after incubation with horseradish peroxidase (HRP)-linked secondary antibody (Cell Signaling Technology, 7074S, Beverly, MA) (1:8,000) using an Amersham ECL western blotting detection reagent (GE Healthcare, Buckinghamshire, UK). Relative band intensity was determined by using the ImageJ software.

### Cell Invasion Assay

After 5 days mono- or co-culture, PBMCs were removed from the co-cultures, and breast cancer cells were detached by trypsin. Following washing in PBS, cell invasion test was performed in the Transwell-24 well with 8-μm pores (SPL Life Sciences, Korea) coated with 60 μl of diluted Matrigel (0.5 mg/ml, BD Biosciences, San Jose, USA) on the inner surface described before. Once the Matrigel was polymerized, mono- and co-cultured breast cancer cell suspensions (50,000 cells/well in a serum-free medium) were placed in the upper chamber and 750 μl of chemoattractant (a complete medium containing 10% FBS) was added to the lower compartment in order to attract cells. In this case, cells could then adhere to or migrate through the porous membrane in response to the chemo-attractant. Upon 24 h incubating at 37°C, non-invaded cells were removed from the top of the Matrigel using a cotton-tipped swab, and the cells on the underside of the membrane were washed and fixed in 4% PFA and stained for 20 min in a crystal violet solution (0.5 mg/ml). Finally, four random fields of the penetrated cells in each well were counted by using a microscope at ×40 magnification in order to assess the invasiveness. Each invasion assay was repeated at least in three independent experiments.

### NF-κB Activity Assay

Five days after mono- or co-culturing of MCF-7 cells with PBMCs, the activity of NFKB was evaluated using a TransAM® NFκB Transcription Factor ELISA Kit (#40096, Active Motif, Belgium) according to the kit's instructions. Following incubation time, cells were washed two times with 1 ml of PBS supplementing with PhosStop phosphatase inhibitor cocktail (Roche, USA) and 0.05% NP-40. An ultracentrifugation was applied to extract nucleus from the cytoplasmic fraction. Upon lysing the nucleus pellet by nucleus lysis buffer and further centrifugation at 14,000× g to extract nuclear proteins, protein concentration was determined using Bradford assay. Ten micrograms of nuclear extract was added to each well of DNA-coated wells. To measure the fraction of DNA-attached NF-kB, HRP-conjugated anti-phospho-p65 primary antibody was then added to each well, and the fluorescence intensity was determined at 630 nm (Stat Fax-2100, ST. Louis, USA).

### LC-MS/MS

A quantitative LC-MS/MS was carried out in order to screen both proteome and secretome profiles of the PBMCs co-cultured with MCF-7 cells compared to those of the untreated PBMCs obtained from patients characterized as hormone and Her2 receptors positive. The experimental design included samples from patients with the confirmed breast cancer patients (stage 2/3). Experiments were performed in pooled biological replicates.

### Sample Preparation for LC-MS/MS

Total proteins were extracted from the co-cultured PBMCs and PBMCs alone using the lysis buffer mentioned earlier, followed by SDS sedimentation using 1 M of KCl solution. Proteins were resuspended in a cocktail containing urea (6 M) + thiourea (2 M) + CHAPS (4%) + 50 mM Tris pH 8.0 and then quantified using Pierce 660-nm protein assay. A 40 μg protein sample was reduced with 10 mM of DTT for 15 min at 65°C and then alkylated for 30 min at room temperature in the dark using 15 mM of iodoacetamide. Afterwards, proteins were precipitated overnight by adding eight volumes of ice-cold acetone plus one volume of ice-cold methanol and incubated at −80°C overnight. Proteins were pelleted by centrifugation, and the pellet was then washed three times with ice-cold methanol. Finally, proteins were resuspended in 100 μl of 0.75 M urea plus 50 mM Tris pH 8 and digested using 1 μg of Trypsin/LysC overnight at 37°C with agitation. Samples were then acidified with 2% of formic acid, and the peptides were purified by the reversed phase SPE (solid phase extraction).

### LC-MS/MS Parameters (MRM)

Acquisition was performed with a Triple TOF 5600 (ABSciex, Foster City, CA, USA) equipped with an electrospray interface with a 25 μm i.d. capillary and coupled to an Eksigent μUHPLC (Eksigent, Redwood City, CA, USA). Analyst TF 1.7 software was applied to control the instrument, process, and acquire the data. Acquisition was performed in SWATH acquisition mode. For the SWATH mode, the source voltage was set to 5.5 kV and maintained at 225°C. Curtain gas was established at 25 psi; ion gas one at 16 psi and ion gas two at 15 psi. Separation was performed on a reversed phase HALO C18-ES column (0.3 mm i.d., 2.7-μm particles, 150 mm long; Advance Materials Technology, Wilmington, DE), which was maintained at 60°C. Samples were injected by loop overfilling into a 5-μl loop. For the 60-min LC gradient, the mobile phase consisted of the following solvents: A (0.2% v/v formic acid and 3% DMSO v/v in water) and B (0.2% v/v formic acid and 3% DMSO in EtOH) at a flow rate of 3 μl/min.

### Data Quantification

Samples were quantified using the SWATH atlas human ion library with the PeakView software (Sciex). A peptide was considered as adequately integrated if it had a score higher than 1.5 or an FDR lower than 0.05. The sum of each adequately integrated peptide was computed for each protein with a max of 15 peptides per protein.

### Identification of the Differentially Expressed Proteins (DEPs) and Bioinformatics Analysis

Proteins were quantified and normalized from two pooled biological replicates. The Log2 fold change value was utilized to select the differentially expressed proteins. Log2 fold changes with a cutoff value of 0.6 were considered to be significant. Among these, those with a log2 ratio >0.6 were regarded as upregulated and those < −0.6 as downregulated proteins. The differentially expressed proteins were further subjected to protein–protein interaction (PPI) and functional analyses.

The STRING software (version 11.0; https://string-db.org/) was applied to construct PPI networks using the default parameters and confidence levels (0.4). The Cytoscape software (version 3.5.1) was used for network visualization. Analysis for topological properties of the network with a different criterion like degree of connectivity to a node was calculated using Network Analyzer plugin algorithms in the Cystoscope in which nodes were ranked according to the degree. Gene ontology and pathway enrichment analysis of the differentially expressed proteins in the proteome and the secretome of the PBMCs was carried out using the Enrichr web tool (https://amp.pharm.mssm.edu/Enrichr/) to detect proteins behind the similar processes. An enrichment *p*-value calculation based on the modified Fisher's exact test was done for the most relevant biological terms enriched in the given list. The enrichment *p*-value was corrected using the Benjamin–Hochberg method to control the false discovery rate. Moreover, the SecretomeP web tool was used to identify the PBMCs-secreted proteins (http://www.cbs.dtu.dk/services/SecretomeP/).

### RNA Extraction and Quantitative Real-Time PCR (qPCR) Analysis

To further confirm the identified proteins in PBMCs, their mRNA levels were examined in co-cultured PBMC samples along with PBMCs isolated from another set of 10 metastatic breast cancer patients using the quantitative real-time PCR assay. Briefly, after co-culturing, total mRNA was extracted from PBMCs using RNX-Plus (Cat. No: YT9066-YT9064, YTA, Iran) according to the manufacturer's instructions. RNA concentration and purity were spectrophotometrically quantified based on measurement of the absorbance at 280 nm. First strand complementary DNA (cDNA) was synthesized using 542 ng of total mRNA by a cDNA synthesis Kit with MMLV reverse transcriptase (Cat No: YT4500, YTA, Iran). Data were normalized to the GAPDH expression level and presented as the averages from two independent experiments. The primer sequences of the target genes are listed in Table 1.

**Table 1:** Sequences of the primers used for detection and quantification of the mRNA expression level.

**Applications**	**Primers**	**Primer sequence (5′ → 3′)**
RT-PCR	F_CANX	ATACCACTGCTCCTCCTTCATC
	R_CANX	TACCTCCCACTTTCCATCATATTTG
RT-PCR	F_CUL4A	AATTGCTTATATGCTGCCGAAGG
	R_CUL4A	AGTGGTTTCTGTGTGCTGTGG
RT-PCR	F_HSPA4	ACAGGATTAACAGGTATAAAGGTGAC
	R_HSPA4	AGGAACCGAAACAACACAGTC
RT-PCR	F_S100A9	CTCCTCGGCTTTGACAGAGTG
	S_S100A9	TATTGGTGGAAGGTGTTGATGATGG
RT-PCR	F_SRSF6	GCCTATCCTTGCCTGTAGTTCTC
	R_SRSF6	TTTATCAGTCAACCCTATGCTCACC
RT-PCR	F_THBS1	AGCATCCGCAAAGTGACTGAAG
	R_THBS1	CTGAACTCCGTTGTGATAGCATAGG
RT-PCR	F_TMSB4X	TGACAAACCCGATATGGCTGAG
	R_TMSB4X	CTGCTTGCTTCTCCTGTTCAATC
RT-PCR	GAPDH-F	ACATCAAGAAGGTGGTGAAGCAG
	GAPDH-R	GCGTCAAAGGTGGAGGAGTG

### Prognostic Impact of the Identified Proteins in Breast Cancer Patients With High and Low Expression Levels

Survival rates were estimated by the Kaplan–Meier method using the web tool available at https://kmplot.com/. This tool performs validation and prognostic analysis of the available gene expression datasets. The hazard ratio (HR) was used to predict the impact of genes on the survival rate. *P* < 0.05 was considered as statistically significant.

### Statistical Analysis

Assays were conducted in at least three independent experiments to obtain data for statistical analyses. No statistical method was used to calculate sample size. For Western blotting data, representative images from three biological replicates are shown. Statistical differences were determined by two-tailed unpaired Student's *t*-test between two groups or ANOVA among more than two groups, as appropriate. Statistical analysis was performed using GraphPad Prism 7. A *p*-value of 0.05 was considered as statistically significant. The levels of mRNA expression were established by a linear model. Receiver operating characteristic (ROC) curves and the area under the curves (AUCs) were constructed to evaluate the diagnostic values of each biomarker. The Youden index was determined using the DiagTest3Grp package in R software (30). All quantitative data are presented as mean ± standard errors (SEM).

## Results

### PBMCs Induced EMT in Breast Cancer Cells

**Figure 1:**
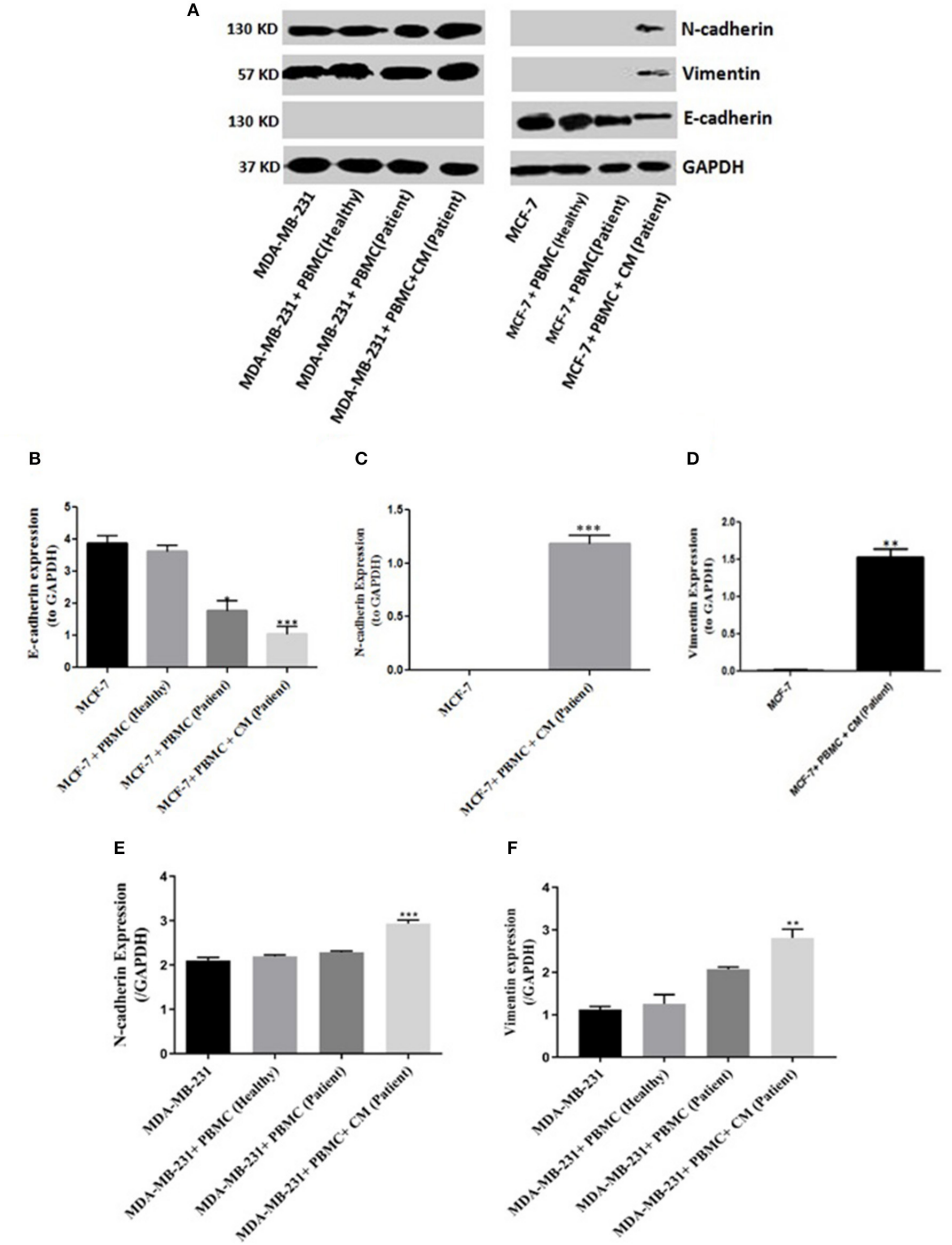
EMT markers expression in breast cancer cells. Immunoblot analysis illustrates expression of various epithelial and mesenchymal markers in breast cancer cells: **(A)** MCF-7 cells (right column) as well as MDA-MB-231 cells (left column). **(B–F)** Quantification of the intensity using the ImageJ software. Data are representative of at least three independent experiments with three biological replicates (*p < 0.05, **p < 0.01, ***p < 0.001).

To evaluate whether PBMCs are able to induce EMT in the epithelial-like or mesenchymal-like breast cancer cells, we co-cultured freshly isolated PBMCs with two human breast cancer cell lines (MCF-7 and MDA-MB-231) exhibiting two different patterns of E-cadherin and N-cadherin expression for 5 days. We then assessed the EMT-related surface marker changes in the breast cancer cell lines following co-culturing. Since no changes in the expression levels of EMT markers were detected in the two cell lines after co-culturing with the PBMCs taken from healthy individuals, we decided to further examine the breast cancer cell lines co-cultured with PBMCs obtained from patients in the primary stage of breast cancer. As shown in Figures 1A–D, PBMCs from breast cancer patients decreased E-cadherin expression in MCF-7 cells. This effect, though somewhat stronger, was also observed with 50% of MCF-7 condition media (CM). Interestingly, an elevated expression level of mesenchymal proteins (N-cadherin and vimentin) was detected only in the presence of CM. MDA-MB-231 cells revealed a significant increase in vimentin and N-cadherin expression upon co-culturing with PBMCs obtained from triple negative patients in the mere presence of conditioned media (50%) (Figures 1E,F). Of note, we detected no E-cadherin expression in MDA-MB-231 cells before and after co-culturing with PBMCs, observations that were in line with previous studies reporting that the E-cadherin expression level in MDA-MB-231 cells is suppressed by methylation in the promoter of the gene (31, 32). Our findings strengthen the hypothesis that PBMCs might be involved in EMT induction in premalignant breast cancer cells to acquire a metastatic phenotype, which acts as an undesirable factor to activate cancer invasion (25, 33).

### PBMCs Enhanced Invasiveness of Breast Cancer Cells

**Figure 2:**
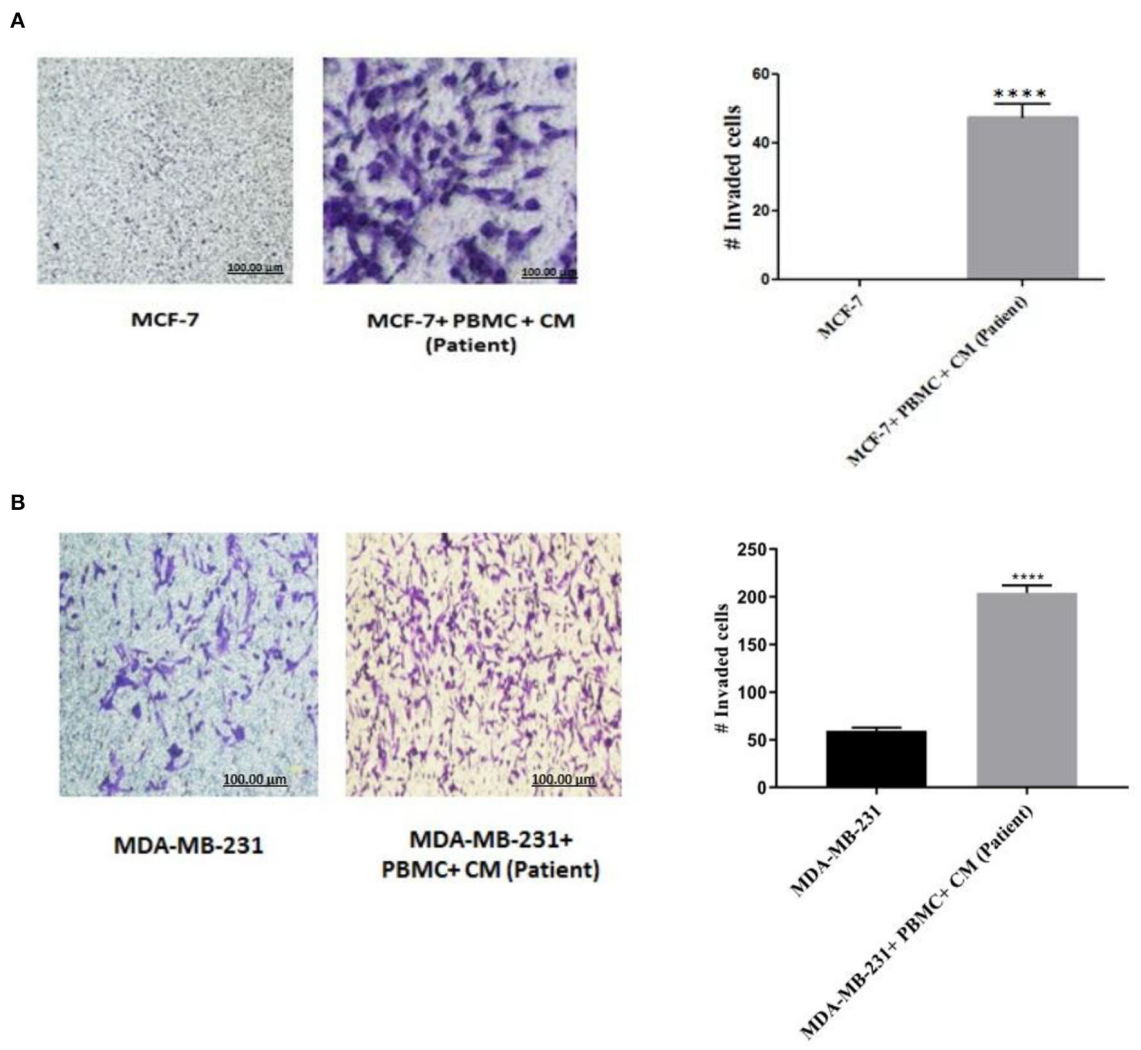
Matrigel invasion assay of **(A)** MCF-7 and **(B)** MDA-MB-231 cells after co-culturing with PBMCs in the presence of 50% conditioned media (CM) for 5 days. Results reported as mean ± SEM performed in triplicates, ****p < 0.0001; *p-*values were obtained using two-tailed Student's *t*-tests.

To confirm that the expression of EMT markers correlates with an increased invasive behavior in the breast cancer cells upon co-culturing with PBMCs, matrigel invasion assay was applied. As shown in Figure 2A, in the absence of PBMCs, no MCF-7 cells invaded the membrane. Albeit, a strong induction of cell invasion was visualized in the presence of PBMCs. Furthermore, co-culturing with PBMCs elevated the number of invaded MDA-MB-231 cells by about 4-fold compared to the MDA-MB-231 cells alone with a partial aggressive phenotype (Figure 2B). These data support the findings of EMT marker determination and demonstrate the strong effect of PBMCs on the invasiveness of breast cancer cells.

### NF-κB Promoted Breast Cancer Cell Invasion

**Figure 3:**
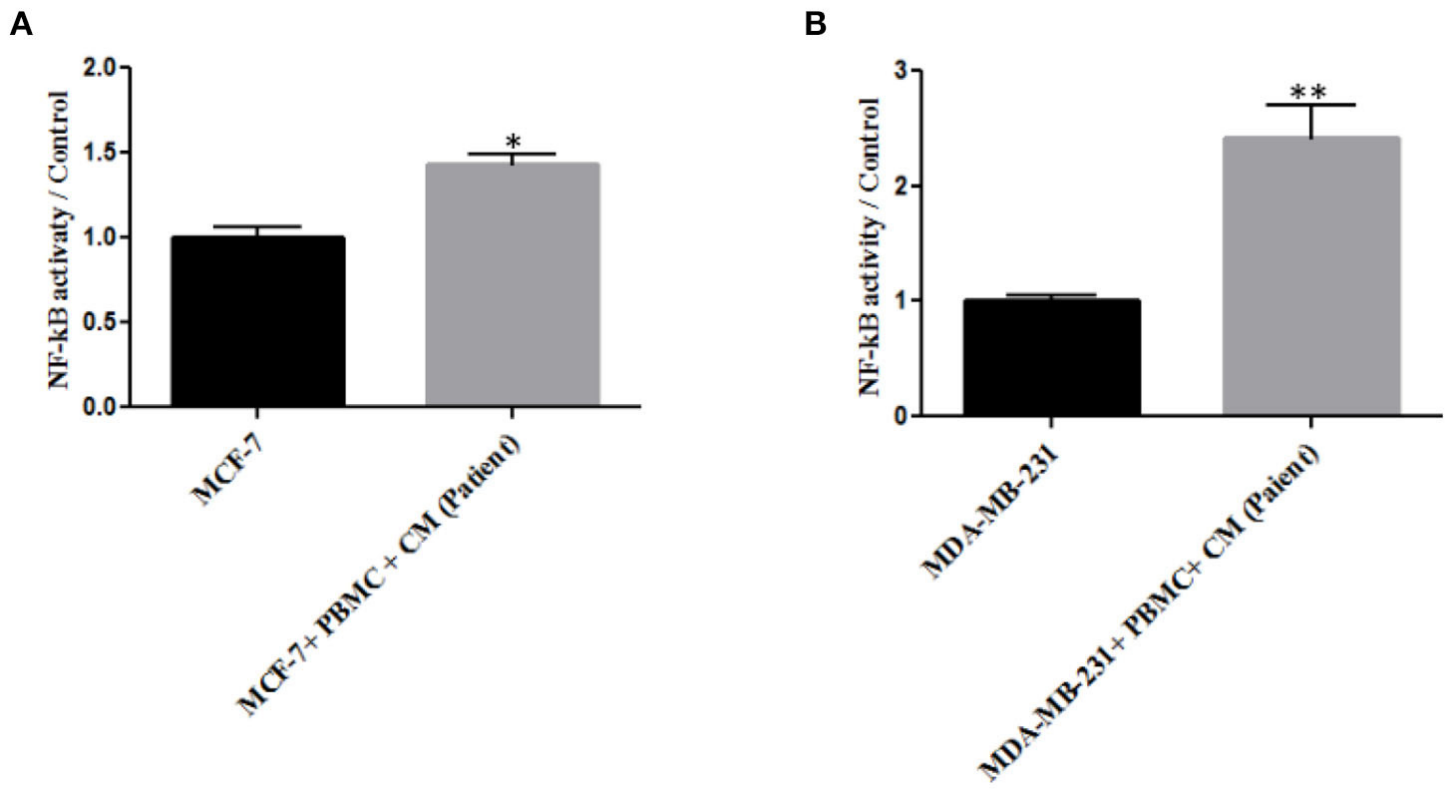
The relative level of NF-kB activity in **(A)** MCF-7 and **(B)** MDA-MB-231 breast cancer cells. Data are represented as mean ± SEM performed in triplicates (*p < 0.05, **p <0.01). *p*-values were obtained using two-tailed Student's *t*-tests.

As NF-κB is an essential transcription factor for inducing EMT through different pathways (25, 34), we thus decided to explore the role of PBMCs in EMT induction by determining NF-κB transcriptional activity. A p65 nuclear translocation in both MCF-7 and MDA-MB-231 cells co-cultured with PBMCs was revealed. As illustrated in Figure 3, PBMCs enhanced NF-κB transcriptional activity by about 1.4- and 2.2-fold in MCF-7 and MDA-MB-231 cells, respectively, compared to the breast cancer cells alone. Since NF-κB activity is highly associated with the ability to secrete a wide panel of cytokines leading to immune cell differentiation (25), we also analyzed the secretome of the breast cancer cells before and after co-culturing with PBMCs.

### Proteome and Secretome Profiles of PBMCs

One of the goals in this study was to evaluate the prognostic and diagnostic values of PBMCs in breast cancer patients with the assumption that a proper protein signature might be identified in the associated PBMCs. Hence, we performed SWATH mass spectrometry–based label free quantitative analysis of the proteins extracted from the PBMCs before and after co-culturing with MCF-7 cells. A total of 440 proteins were identified in both co-cultured PBMCs and PBMCs alone, of which 290 belonged to the former and 393 to the latter (the sum of each adequately integrated peptide was calculated for each protein with a maximum of 15 peptides per protein) (FDR < 0.05). Fold change (≤-1.5 and ≥1.5) of proteins between the co-cultured and control groups was established as a criterion to categorize the differentially expressed proteins (Supplementary Table 1). As a result, among the 222 differentially expressed proteins from the 440 detected proteins, 164 and 58 were up- and downregulated in the co-cultured PBMCs compared to the control, respectively. Furthermore, to identify the mediators involved in EMT-associated alterations induced by PBMCs, supernatants of mono- and co-cultured PBMCs and MCF-7 cells were also subjected to mass spectrometry analysis. Out of the 308 identified proteins, 137 belonged to the supernatant sample of the co-cultured PBMCs, 114 to PBMCs alone, and 105 to MCF-7 cells. Among the differentially expressed proteins in the supernatant of the co-cultured PBMCs vs. PBMCs alone, 124 were upregulated and 10 downregulated. Besides, of the 128 differentially expressed proteins in the supernatant of the co-cultured PBMCS, 116 were high-abundant and 12 were low-abundant compared to the supernatant of the MCF-7 cells alone. These results are presented in Supplementary Table 1.

### Bioinformatics Analysis

In order to achieve a global insight into the differentially expressed proteins associated with EMT, Gene Ontology and KEGG pathway analysis along with the related diseases and disorders were applied to analyze molecular and cellular functions as well as the canonical pathways altered in PBMCs during the EMT process. Functional protein association networks were constructed and visualized using the String database and the Cytoscape software.

GO analysis was used to assign the functional relevance of the differentially expressed proteins in the proteome and secretome of PBMCs to three independent ontologies: biological processes (BPs), molecular functions (MFs), and cellular components (CCs). In terms of BPs, 222 altered proteins in the proteome were annotated as being involved in negative regulation of programmed cell death, positive regulation of cell morphogenesis contributing to cell differentiation, positive regulation of NF-kappaB transcription factor activity, glyceraldehyde-3-phosphate metabolic process, antigen processing and presentation of exogenous peptide antigen, pattern recognition receptor signaling pathway, positive regulation of protein kinase B signaling, and glucose 6-phosphate metabolic process (Table 2). With respect to MF ontology, the key functions of the altered proteins were linked to binding, such as RNA binding (GO:0003723), cadherin binding (GO:0045296), actin binding (GO:0003779), RAGE receptor binding (GO:0050786), and protein kinase binding (GO:0019901). Furthermore, the most enriched cellular components were related to the secretory granule lumen (GO:0034774), focal adhesion (GO:0005925), cytoskeleton (GO:0005856), ficolin-1-rich granule lumen (GO:1904813), actin cytoskeleton (GO:0015629), lysosome (GO:0005764), lytic vacuole (GO:0000323), nucleolus (GO:0005730), and INO80-type complex (GO:0097346). Pathway analysis of the differentially expressed proteins in the co-cultured PBMCs vs. PBMC control cells revealed PPAR, IL-17, and PI3K-Akt as the most highly enriched signaling pathways, most likely involved in cancer progression including breast cancer. In addition, other pathways such as proteoglycans in cancer, pentose phosphate, phagosome, pathogenic *Escherichia coli* infection, and bacterial invasion of epithelial cells were also enriched (Table 3). These results imply the interplay of inflammatory and metabolic pathways in cancer progression during the EMT process.

**Table 2:** GO enrichment analysis (biological process) of the proteome of co-cultured PBMC vs. PBMC alone.

**Biological process name**	***P*-value**	**FDR**	**#Entities found**	**#Entities total**
Negative regulation of programmed cell death (GO:0043069)	3.47E-06	9.85E-04	17	17
Antigen processing and presentation of exogenous peptide antigen (GO:0002478)	1.36E-05	0.002662	8	8
Positive regulation of NF-kappaB transcription factor activity (GO:0051092)	1.41E-05	0.002566	9	9
Glyceraldehyde-3-phosphate metabolic process (GO:0019682)	4.00E-05	0.005834	4	4
Positive regulation of cell morphogenesis involved in differentiation (GO:0010770)	4.82E-05	0.00665	6	6
Pattern recognition receptor signaling pathway (GO:0002221)	1.82E-04	0.022693	5	5
Glucose 6-phosphate metabolic process (GO:0051156)	6.79E-04	0.045002	3	3
Positive regulation of protein kinase B signaling (GO:0051897)	5.73E-04	0.04241	7	7

**Table 3:** Pathway analysis of the proteome of co-cultured PBMC vs. PBMC alone.

**Pathway name**	***P*-value**	**FDR**	**#Entities found**	**#Entities total**
Phagosome	1.08E-06	3.33E-04	11	152
Pathogenic *Escherichia coli* infection	2.44E-06	3.75E-04	7	55
Bacterial invasion of epithelial cells	5.12E-06	0.01	6	74
Complement and coagulation cascades	8.36E-05	0.01	6	79
Proteoglycans in cancer	1.73E-04	0.01	9	201
Protein processing in endoplasmic reticulum	2.47E-04	0.02	8	165
Regulation of actin cytoskeleton	4.25E-04	0.02	9	214
PPAR signaling pathway	5.25E-04	0.03	5	74
Antigen processing and presentation	6.68E-04	0.03	5	77
IL-17 signaling pathway	0.003	0.05	5	93
Pentose phosphate pathway	0.004	0.05	3	30
PI3K-Akt signaling pathway	0.044	0.34	8	354

Moreover, following BP analysis of the supernatant of the co-cultured vs. PBMC control cells, 181 differentially expressed proteins were significantly enriched, based on a *P*-value of <0.05, in processes including nuclear transport (GO:0051169), regulation of DNA-templated transcription in response to stress (GO:0043620), superoxide metabolic processes (GO:0006801), signal transduction involving mitotic G1 DNA damage checkpoint (GO:0072431), nucleotide-excision repair (GO:0006289), DNA damage response, and signal transduction by a p53 class mediator resulting in cell cycle arrest (GO:0006977).

Pathway analysis of the secretome from the co-cultured PBMCs vs. control PBMCs identified 44 significantly enriched pathways based on the *P* < 0.05, the most prominent of which were as follows: oocyte meiosis, cell cycle, glioma, prion diseases, starch and sucrose metabolism, thyroid cancer, bladder cancer, PI3K-Akt signaling pathway, human immunodeficiency virus 1 infection, human T-cell leukemia virus 1 infection, and HIF-1 signaling pathway. Enrichment of these pathways is in agreement with the signaling pathways of the proteome highlighting the EMT in breast cancer cells. Consistently, pathway analysis of the co-cultured supernatants vs. tumor cells exhibited similar results (data are not shown). Analysis of the related diseases and disorders of the altered proteins in the proteome of the co-cultured PBMCs vs. PBMCs alone unveiled a contribution of these proteins in the tumor progression of various cancers including liver carcinoma, metastatic breast cancer, breast carcinoma, adenocarcinoma of the lung, and malignant neoplasm of the stomach.

**Figure 4:**
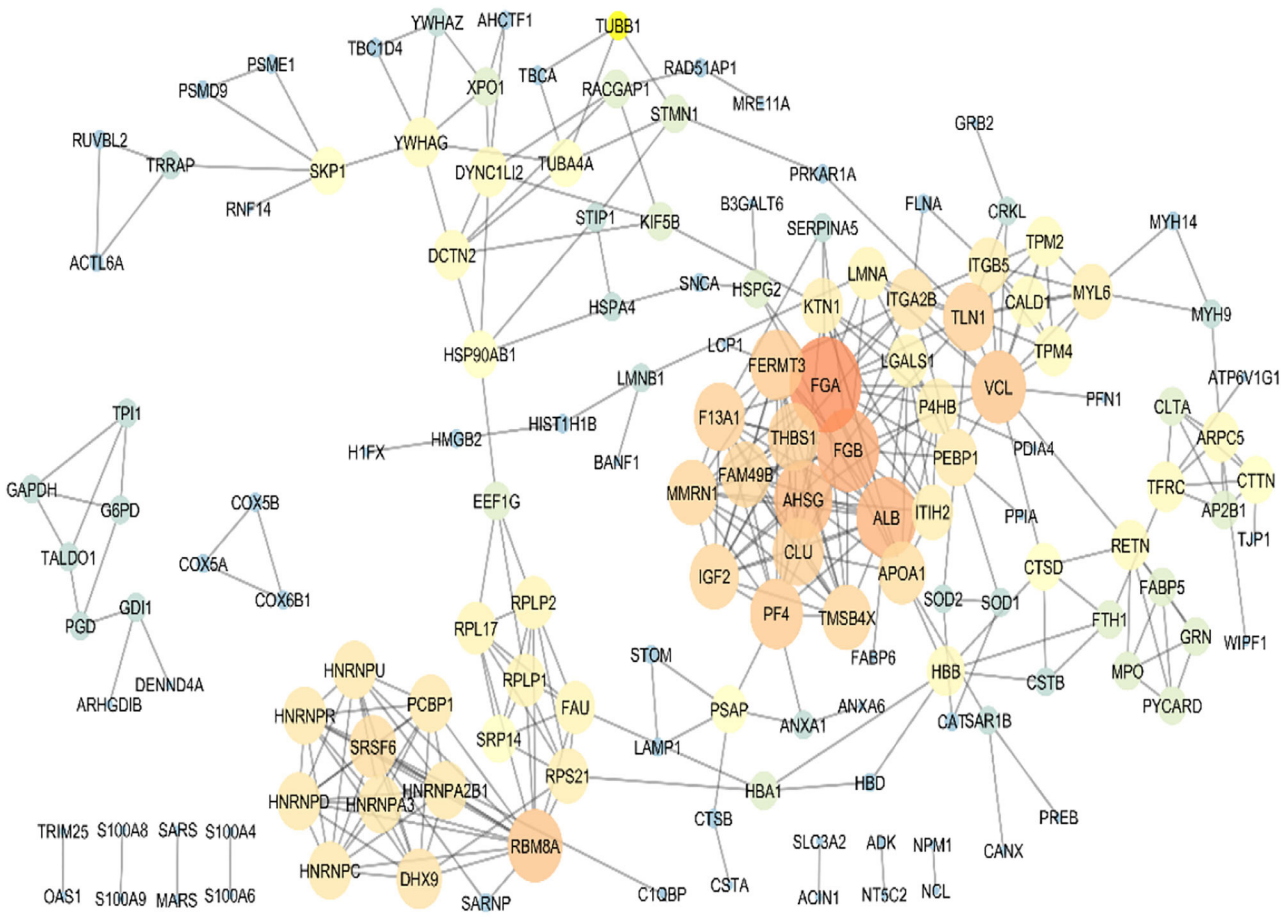
Protein–protein interaction map of the differentially expressed proteins in the co-cultured PBMCs vs. PBMCs alone. Major hubs were highlighted. TMSB4X, SRSF6, and THBS1 are represented as significant signature based on the high connectivity degree value in the human protein–protein interaction networks.

To sum up, based on the bioinformatics analysis, seven target proteins were subjected to further analysis in the PBMC samples of metastatic and non-metastatic cancer patients. These proteins included TMSB4X, HSPA4, S100A9, SRSF6, THBS1, CUL4A, and CANX. PPI analysis of the co-cultured PBMCs proteome was performed for protein rank determination (Figure 4). TMSB4X, SRSF6, and THBS1 displayed high connectivity degree values among the human protein–protein interaction networks according to the topological analysis, introducing these proteins as potential signatures in PBMCs of breast cancer patients.

### Further Confirmation of Data Using Quantitative Real-Time PCR (qPCR)

**Figure 5:**
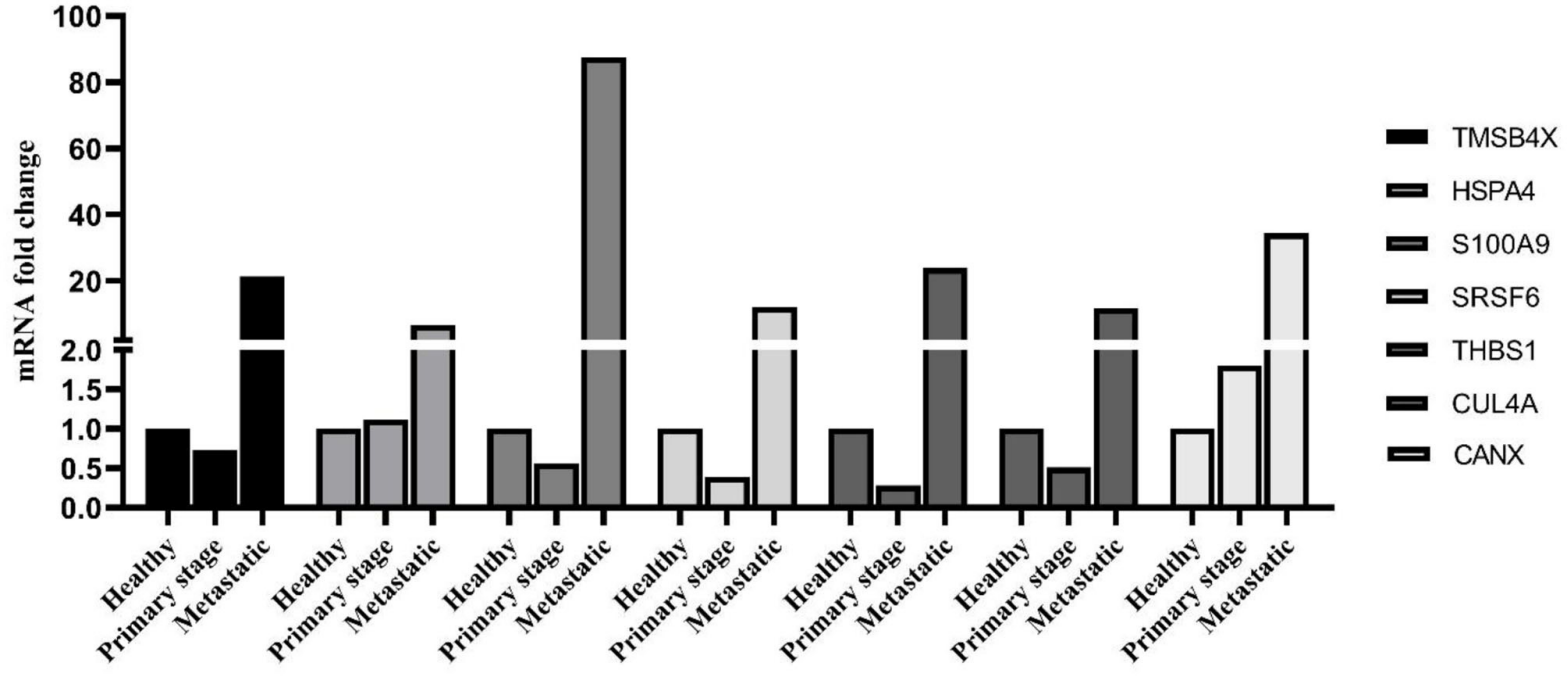
mRNA expression level of PBMC signature genes between different groups. The linear model was used to determine statistical significance at the level of *p* < 0.05.

As shown in Figure 5, the identified PBMC biomarkers (TMSB4X, HSPA4, S100A9, SRSF6, THBS1, CUL4A, and CANX) were significantly upregulated in the breast cancer patients at the metastatic stage compared to both the primary stage and healthy individuals (*p* < 0.001). The mRNA levels of SRSF6 and CUL4A were significantly decreased in patients at the primary stage in comparison with those in the healthy individuals (*p* < 0.001).

**Figure 6:**
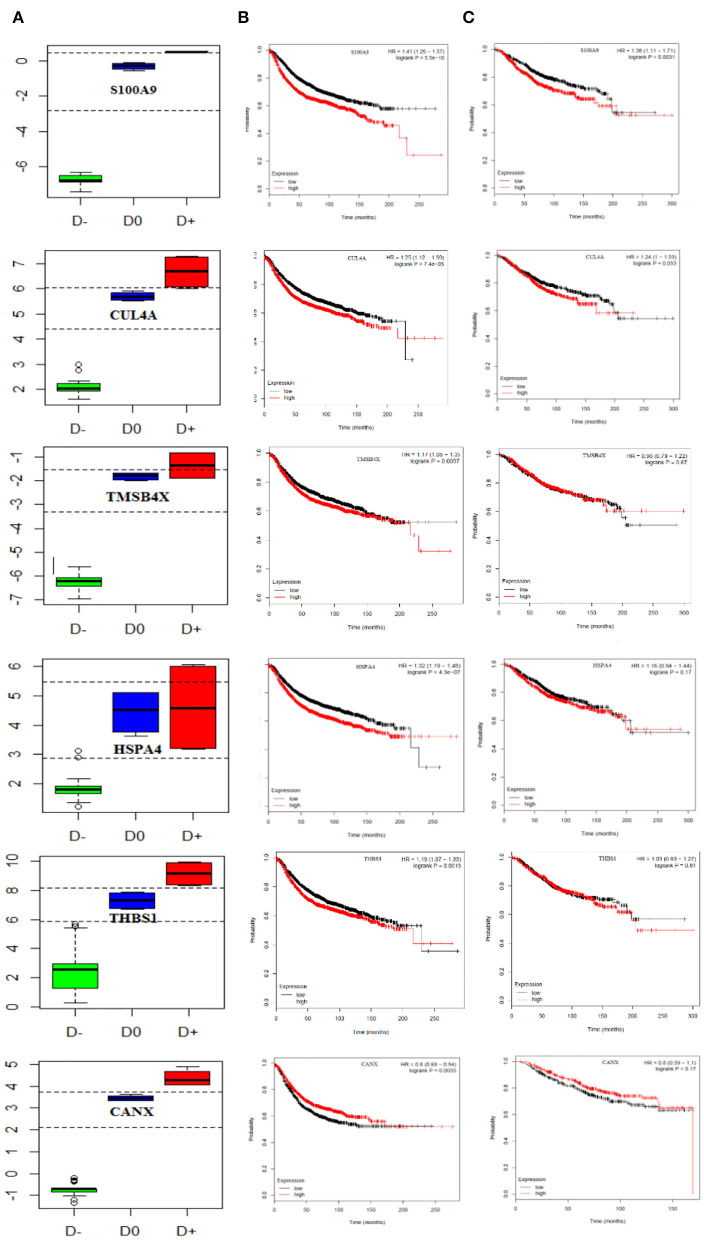
Prognostic curve of the related PBMC gene signatures. **(A)** Boxplot of the Youden index calculated for PBMC gene signatures, S100A9, SRSF6, THBS1, CUL4A, and CANX, could accurately distinguish metastatic and primary breast cancer patients from healthy objects. D^−^ refers to metastatic breast cancer patient, D^0^ healthy subjects, and D^+^ primary breast cancer patient cut-points (optimal cut-points indicated in dashed lines). Kaplan–Meier curves show **(B)** relapse-free survival in breast cancer patients and **(C)** overall survival and (TCGA data set). The red and black curves represent the high and low expression levels, respectively. *p*-values are shown at the upper-right corner. HR, hazard ratio.

We also performed multivariate analysis across the identified biomarker list to explore the best biomarker candidates. Our analysis introduced CANX with the highest diagnostic value in distinguishing breast cancer patients at the primary or metastatic stages from the healthy individuals. To further verify the diagnostic value of CANX in breast cancer patients, receiver-operating characteristic (ROC) curve analysis was applied. The results indicated that AUC, sensitivity, and specificity were 100% for CANX (Supplementary Figure 1). Youden index analysis also showed that five proteins including S100A9, SRSF6, THBS1, CUL4A, and CANX can accurately diagnose breast cancer patients at the metastatic and primary stages from healthy individuals (Figure 6A).

Finally, we attempted to evaluate the prognostic impact of this set of genes as proliferation markers on the survival rate of breast cancer patients. To do this, survival rates were measured using the Kaplan–Meier method, and the results are illustrated in Figures 6B,C. Our findings revealed increased levels of S100A9 and CUL4A leading to a reduced overall and relapse-free survival rate in breast cancer patients. CANX showed no significant impact on either the overall or relapse-free survival rate (hazard ratio = 0.8, *P* = 0.17).

## Discussion

It has repeatedly been reported that transcriptome profiling in PBMCs is altered in the context of cancers (33, 35). Formation of metastasis occurs due to not only epithelial to mesenchymal transition but also the immunomodulatory effect of tumor cells on PBMCs allowing cancer cells to escape the immune attack (33). Consequently, investigation on PBMC profile has drawn a great deal of attention among researchers. Moreover, PBMC protein profile represents a reflection of the proteins in tumor cells, providing a possibility for the prediction of tumor behavior, patient outcome, and making treatment decisions. Taking into account the prognostic impact of PBMCs in breast cancer, we decided to enhance our understanding of the molecular biology of PBMCs in order to offer new perspectives on breast cancer metastasis. Our current findings indicated that co-culturing with PBMCs promotes the invasiveness of breast cancer cells *in vitro* and enhances NF-kB activity as well, suggesting the potential role of PBMCs in the progression of breast cancer. Hence, PBMC protein profiling was evaluated to discover the underlying mechanisms.

Next, we implemented SWATH MS-based biomarker discovery and identified 440 proteins in the co-cultured PBMCs and PBMCs alone. Our findings revealed that co-cultured PBMCs express proteins involved in breast cancer progression, a finding in line with our previous *in vitro* results. Functional annotation and enrichment analysis of 222 differentially expressed proteins with a greater than 1.5-fold change indicated that co-culturing with breast cancer cells leads to immunosuppression of PBMCs by modulating several specific tumor progression pathways (i.e., PPAR, IL-17, PI3K-Akt, positive regulation of protein kinase B signaling, and HIF-1 signaling pathway, positive regulation of NF-kB transcription factor activity). In addition, based on the functional analysis of PBMC proteome, we identified a set of seven well-known tumor progression proteins including TMSB4X, HSPA4, S100A9, SRSF6, THBS1, CUL4A, and CANX, which take a part in the above-mentioned pathways.

In order to give an explanation for the high- and low-abundant proteins, we point out some roles of these proteins in cancer progression. In this regard, Thymosin-ß4 (Tß4; TMSB4X) and THBS1 are considered as tumor promoters with a poor prognosis in different types of cancers (25–28). Interestingly, TMSB4X has a function in the TGF-ß/Tß4/MRTF signaling pathway, leading to EMT induction and metastasis (36). SRSF6 behaves as an oncogene protein and is associated with proliferation, transformation, and tumorigenicity of immortal cancer cells (37–40). Of note, our network analysis of PBMC proteome established these three proteins as a significant signature based on the topological characteristics displaying a high connectivity degree value among the human protein–protein interaction networks.

As described in the literature, HSPA4 is a tumor membrane antigen having a role in tumor metastasis through activating the NF-κB pathway. Clinically, high HSPA4 expression in breast cancer has been shown to be correlated with increased lymph node metastasis (41). Besides, S100A9 is a member of the calcium-binding protein family, which regulates the inflammatory responses (42). S100A9 is considered as a driving force for migration and invasion in cancer cells through a number of signaling pathways including ERK1/2, MAPK, JNK, and NF-kB (43). Interestingly, S100A9 is expressed in both tumor and infiltrating immune cells. In breast cancer cells, this protein facilitates interactions between tumor cells and their surrounding microenvironment, which results in the aggressive tumor phenotypes and poor survival outcome (43). Cullin4A (Cul4A) as an oncogene (44) mediates the EMT process in breast cancer cells, which in turn causes metastasis by modifying the regulatory ZEB1 gene (45). CANX contributes to the synthesis and folding of proteins through the Calreticulin/CANX cycle (46). CANX expression is significantly correlated with the transition from the angiogenesis-independent to angiogenesis-dependent (i.e., more invasive) tumor growth (47). Overall, these observations imply that PBMCs contain abundant data about the interactions between the tumor and its microenvironment, introducing them as suitable candidates for use as biomarkers in predicting the risk of cancer progression. However, since these findings are solely based on the information obtained from our *in vitro* co-culture data, we next decided to validate the candidate PBMC biomarkers in breast cancer patients.

In the second part of this study, we examined whether the set of seven proteins identified as candidate PBMC biomarkers is expressed in the PBMC fraction of breast cancer patients at primary and metastatic stages. Surprisingly, most of the proteins did not exhibit the same expression patterns detected in either the co-cultured samples or in the samples at different stages of breast cancer. Furthermore, our findings revealed opposing abundance levels of some of the identified proteins in the PBMCs of primary and metastatic BC patients; HSPA4 and CANX had a significantly high abundance level in patients at both primary and metastatic stages, while the remaining proteins were downregulated in the patient's PBMCs at the primary stage, which were in contrast to the metastatic stage.

Interestingly, previous results have demonstrated a correlation between the expression of immune-related genes in PBMCs and a downregulated expression in tumor tissues (35, 48, 49). In line with these reports, our findings also revealed downregulation in PBMCs of TMSB4X, SRSF6, THBS1, CUL4A, and S100A9 genes in patients at the early stage, suggesting an inverse correlation between their expression in tumor tissues and PBMCs of the primary stage patients. Of particular note is the similarity between the expression pattern of these genes in patients at the metastatic stage and those obtained from tumor tissues.

Here, the question then arises as to whether the PBMCs overexpress these genes or they capture the tumor-derived exosomes leading to the high expression levels of these genes in PBMCs. Our ongoing research is focused on evaluating the role of exosomes as metastatic material carriers in the immunomodulation of PBMCs.

Additionally, we performed Youden index analysis in order to identify a biomarker that is capable of differentiating healthy individuals from BC patients at primary and metastatic stages. Our data revealed that except for TMSB4X and HSPA4, the remaining genes can be regarded as suitable candidates. Moreover, in order to explore the effective biomarkers for diagnosis, multivariate analysis was carried out revealing CANX as the only candidate with the highest diagnostic efficiency in distinguishing metastatic from primary BC patients and healthy objects. Further verification through ROC analysis revealed significant sensitivity and specificity for CANX, introducing it as a protein with a promising diagnostic value. In order to evaluate the prognostic aspect of the identified genes, Kaplan–Meier analysis using a publicly available dataset was carried out. Among the seven genes examined, a high expression level of S100A9 was associated with a significantly shorter overall and relapse-free survival in breast cancer patients. In other words, although CANX could be considered as a suitable diagnostic PBMC marker, its prognostic effect depends on the subtype of breast cancer.

In summary, the present study implies the immunosuppressive role of PBMCs in tumor progression of breast cancer cells. Indeed, the protein expression profile of PBMCs was a reflection of the proteins expressed in the BC tissues; however, the abundance levels were different due to the stage of the cancer. Furthermore, a gene set consisting of five genes was found to be helpful in distinguishing metastatic from non-metastatic breast cancer patients. Collectively, these results reflect the advantages and major bottlenecks in biomarker discovery using a fully proteomic approach and should be interpreted with caution due to the small number of breast cancer patients evaluated.

## Data Availability Statement

The raw data supporting the conclusions of this article will be made available by the authors, without undue reservation, to any qualified researcher.

## Ethics Statement

The studies involving human participants were reviewed and approved by Human Ethics Research Committee of Shahid Beheshti University of Iran. The patients/participants provided their written informed consent to participate in this study.

## Author Contributions

RM contributed in designing and the development of methodology, data analysis, interpretation of data, and wrote the manuscript. SS, FS, and AM provided the patient consent and collected blood and clinical data. AG and SA helped in the validation of the data. MS was the principal investigator who supervised the study. All authors contributed to the article and approved the submitted version.

## Conflict of Interest

The authors declare that the research was conducted in the absence of any commercial or financial relationships that could be construed as a potential conflict of interest.

## Supplementary Material

Supplementary Table 1 Up- and downregulated proteins in co-cultured PBMC proteome relative to PBMC control, and up- and downregulated proteins in co-cultured PBMC secretome relative to PBMC control and breast cancer cells (MCF-7) (20.0 kB xlsx).

Supplementary Figure 1 The ROC analysis of CANX to detect the metastatic from non-metastatic stages of breast cancer patients. The AUC of CANX was 1 (*p* < 0.05).

## References

[1] Kotiyal S Bhattacharya S. Breast cancer stem cells, EMT and therapeutic targets. Biochem Biophys Res Commun. (2014) 453:112–6. 10.1016/j.bbrc.2014.09.069 25261721

[2] Little AC Pathanjeli P Wu Z Bao L Goo LE Yates JA. IL-4/IL-13 stimulated macrophages enhance breast cancer invasion via Rho-GT pase regulation of synergistic VEGF/CCL-18 signaling. Front Oncol. (2019) 9:456. 10.3389/fonc.2019.00456 31214501 PMC6554436

[3] Ayla S Karahuseyinogluc S. Cancer stem cells, their microenvironment and anoikis. Crit Rev Oncog. (2019) 24:27–34. 10.1615/CritRevOncog.2018029433 31679217

[4] Gonzalez H Hagerling C Werb Z. Roles of the immune system in cancer: from tumor initiation to metastatic progression. Genes Dev. (2018) 32:1267–84. 10.1101/gad.314617.118 30275043 PMC6169832

[5] Dumauthioz N Labiano S Romero P. Tumor resident memory T cells: new players in immune surveillance and therapy. Front Immunol. (2018) 9:2076. 10.3389/fimmu.2018.02076 30258445 PMC6143788

[6] Kourko O Seaver K Odoardi N Basta S Gee K. IL-27, IL-30, and IL-35: a cytokine triumvirate in cancer. Front Oncol. (2019) 9:969. 10.3389/fonc.2019.00969 31681561 PMC6797860

[7] Sanaei MJ Salimzadeh L Bagheri N. Crosstalk between myeloid-derived suppressor cells and the immune system in prostate cancer: MDSCs and immune system in prostate cancer. J Leuk Biol. (2019) 107:43–56. 10.1002/JLB.4RU0819-150RR 31721301

[8] Weng YS Tseng HY Chen YA Shen PC Al Haq AT Chen LM. MCT-1/miR-34a/IL-6/IL-6R signaling axis promotes EMT progression, cancer stemness and M2 macrophage polarization in triple-negative breast cancer. Mol Cancer. (2019) 18:42. 10.1186/s12943-019-0988-0 30885232 PMC6421700

[9] Tunccan T Duzer S Dilek G Yuksel UM Cetiner H Kilic C. The role of CSE1L expression in cervical lymph node metastasis of larynx tumors. Brazil J Otorhinolaryngol. (2019). 10.1016/j.bjorl.2019.06.010. [Epub ahead of print]. 31383592 PMC9422512

[10] Chen Y Zhang S Wang Q Zhang X. Tumor-recruited M2 macrophages promote gastric and breast cancer metastasis via M2 macrophage-secreted CHI3L1 protein. J Hematol Oncol. (2017) 10:36. 10.1186/s13045-017-0408-0 28143526 PMC5286803

[11] Ashraf Y Mansouri H Laurent-Matha V Alcaraz LB Roger P Guiu S. Immunotherapy of triple-negative breast cancer with cathepsin D-targeting antibodies. J Immunother Cancer. (2019) 7:29. 10.1186/s40425-019-0498-z 30717773 PMC6360707

[12] Walker ND Elias M Guiro K Bhatia R Greco SJ Bryan M Exosomes from differentially activated macrophages influence dormancy or resurgence of breast cancer cells within bone marrow stroma. Cell Death Dis. (2019) 10:59 10.1038/s41419-019-1304-z 30683851 PMC6347644

[13] Cortes M Sanchez-Moral L de Barrios O Fernandez-Acenero MJ Martinez-Campanario MC Esteve-Codina A. Tumor-associated macrophages (TAMs) depend on ZEB1 for their cancer-promoting roles. EMBO J. (2017) 36:3336–55. 10.15252/embj.201797345 29038174 PMC5686549

[14] Mantovani A Marchesi F Malesci A Laghi L Allavena P. Tumour-associated macrophages as treatment targets in oncology. Nat Rev Clin Oncol. (2017) 14:399–416. 10.1038/nrclinonc.2016.217 28117416 PMC5480600

[15] Zhu X Chen L Liu L Niu X. EMT-mediated acquired EGFR-TKI resistance in NSCLC: mechanisms and strategies. Front Oncol. (2019) 9:1044. 10.3389/fonc.2019.01044 31681582 PMC6798878

[16] Inan S Hayran M. Cell signaling pathways related to epithelial mesenchymal transition in cancer metastasis. Crit Rev Oncogen. (2019) 24:47–54. 10.1615/CritRevOncog.2018029509 31679219

[17] Xiao T Jie Z. MiR-21 promotes the invasion and metastasis of gastric cancer cells by activating epithelial-mesenchymal transition. Eur Sug Res. (2019) 60:208–18. 10.1159/000504133 31722341

[18] Benner B Scarberry L Suarez-Kelly LP Duggan MC Campbell AR Smith E. Generation of monocyte-derived tumor-associated macrophages using tumor-conditioned media provides a novel method to study tumor-associated macrophages *in vitro*. J Immunother Cancer. (2019) 7:140. 10.1186/s40425-019-0622-0 31138333 PMC6540573

[19] Noman MZ Janji B Abdou A Hasmim M Terry S Tan TZ. The immune checkpoint ligand PD-L1 is upregulated in EMT-activated human breast cancer cells by a mechanism involving ZEB-1 and miR-200. Oncoimmunology. (2017) 6:e1263412. 10.1080/2162402X.2016.1263412 28197390 PMC5283623

[20] Suarez-Carmona M Lesage J Cataldo D Gilles C. EMT and inflammation: inseparable actors of cancer progression. Mol Oncol. (2017) 11:805–23. 10.1002/1878-0261.12095 28599100 PMC5496491

[21] Zhu H Gu Y Xue Y Yuan M Cao X Liu Q. CXCR2(+) MDSCs promote breast cancer progression by inducing EMT and activated T cell exhaustion. Oncotarget. (2017) 8:114554–67. 10.18632/oncotarget.23020 29383101 PMC5777713

[22] Mantovani A Barajon I Garlanda C. IL-1 and IL-1 regulatory pathways in cancer progression and therapy. Immunological reviews. (2018) 281:57–61. 10.1111/imr.12614 29247996 PMC5922413

[23] Geis-Asteggiante L Belew AT Clements VK Edwards NJ Ostrand-Rosenberg S El-Sayed NM. Differential content of proteins, mRNAs, and miRNAs suggests that MDSC and their exosomes may mediate distinct immune suppressive functions. J Proteome Res. (2018) 17:486–98. 10.1021/acs.jproteome.7b00646 29139296 PMC6200353

[24] Hashimoto O Yoshida M Koma Y Yanai T Hasegawa D Kosaka Y. Collaboration of cancer-associated fibroblasts and tumour-associated macrophages for neuroblastoma development. J Pathol. (2016) 240:211–23. 10.1002/path.4769 27425378 PMC5095779

[25] Su S Liu Q Chen J Chen J Chen F He C. A positive feedback loop between mesenchymal-like cancer cells and macrophages is essential to breast cancer metastasis. Cancer Cell. (2014) 25:605–20. 10.1016/j.ccr.2014.03.021 24823638

[26] Fujisaka Y Iwata T Tamai K Nakamura M Mochizuki M Shibuya R. Long non-coding RNA HOTAIR up-regulates chemokine (C-C motif) ligand 2 and promotes proliferation of macrophages and myeloid-derived suppressor cells in hepatocellular carcinoma cell lines. Oncol Lett. (2018) 15:509–14. 10.3892/ol.2017.7322 29387231 PMC5768083

[27] Deng C Zhang Q Jia M Zhao J Sun X Gong T. Tumors and their microenvironment dual-targeting chemotherapy with local immune adjuvant therapy for effective antitumor immunity against breast cancer. Adv Sci. (2019) 6:1801868. 10.1002/advs.201801868 30937266 PMC6425447

[28] Blomberg OS Spagnuolo L de Visser KE. Immune regulation of metastasis: mechanistic insights and therapeutic opportunities. Dis Model Mech. (2018) 11:dmm036236. 10.1242/dmm.036236 30355585 PMC6215427

[29] Tauriello DVF Palomo-Ponce S Stork D Berenguer-Llergo A Badia-Ramentol J Iglesias M. TGFbeta drives immune evasion in genetically reconstituted colon cancer metastasis. Nature. (2018) 554:538–43. 10.1038/nature25492 29443964

[30] Luo J Xiong C. DiagTest3Grp: an R package for analyzing diagnostic tests with three ordinal groups. J Stat Softw. (2012) 51:1. 10.18637/jss.v051.i03 23504300 PMC3595562

[31] Chao YL Shepard CR Wells A. Breast carcinoma cells re-express E-cadherin during mesenchymal to epithelial reverting transition. Mol Cancer. (2010) 9:179. 10.1186/1476-4598-9-179 20609236 PMC2907333

[32] van Horssen R Hollestelle A Rens JA Eggermont AM Schutte M Ten Hagen TL. E-cadherin promotor methylation and mutation are inversely related to motility capacity of breast cancer cells. Breast Cancer Res Treat. (2012) 136:365–77. 10.1007/s10549-012-2261-8 23053649

[33] Hensler M Vancurova I Becht E Palata O Strnad P Tesarova P. Gene expression profiling of circulating tumor cells and peripheral blood mononuclear cells from breast cancer patients. Oncoimmunology. (2016) 5:e1102827. 10.1080/2162402X.2015.1102827 27141386 PMC4839342

[34] Khan AQ Ahmed EI Elareer NR Junejo K Steinhoff M Uddin S. Role of miRNA-regulated cancer stem cells in the pathogenesis of human malignancies. Cells. (2019) 8:840. 10.3390/cells8080840 31530793 PMC6721829

[35] Ming W Xie H Hu Z Chen Y Zhu Y Bai Y. Two distinct subtypes revealed in blood transcriptome of breast cancer patients with an unsupervised analysis. Front Oncol. (2019) 9:985. 10.3389/fonc.2019.00985 31632916 PMC6779774

[36] Morita T Hayashi K. Tumor progression is mediated by thymosin-beta4 through a TGFbeta/MRTF signaling axis. Mol Cancer Res. (2018) 16:880–93. 10.1158/1541-7786.MCR-17-0715 29330296

[37] Lal S Allan A Markovic D Walker R Macartney J Europe-Finner N. Estrogen alters the splicing of type 1 corticotropin-releasing hormone receptor in breast cancer cells. Sci Signal. (2013) 6:ra53. 10.1126/scisignal.2003926 23821771

[38] Kong J Sun W Li C Wan L Wang S Wu Y. Long non-coding RNA LINC01133 inhibits epithelial-mesenchymal transition and metastasis in colorectal cancer by interacting with SRSF6. Cancer Lett. (2016) 380:476–84. 10.1016/j.canlet.2016.07.015 27443606

[39] Cohen-Eliav M Golan-Gerstl R Siegfried Z Andersen CL Thorsen K Orntoft TF. The splicing factor SRSF6 is amplified and is an oncoprotein in lung and colon cancers. J Pathol. (2013) 229:630–9. 10.1002/path.4129 23132731

[40] Kim HR Lee GO Choi KH Kim DK Ryu JS Hwang KE. SRSF5: a novel marker for small-cell lung cancer and pleural metastatic cancer. Lung Cancer. (2016) 99:57–65. 10.1016/j.lungcan.2016.05.018 27565915

[41] Gu Y Liu Y Fu L Zhai L Zhu J Han Y. Tumor-educated B cells selectively promote breast cancer lymph node metastasis by HSPA4-targeting IgG. Nat Med. (2019) 25:312–22. 10.1038/s41591-018-0309-y 30643287

[42] Zhang S Wang Z Liu W Lei R Shan J Li L. Distinct prognostic values of S100 mRNA expression in breast cancer. Sci Rep. (2017) 7:39786. 10.1038/srep39786 28051137 PMC5209742

[43] Miller P Kidwell KM Thomas D Sabel M Rae JM Hayes DF. Elevated S100A8 protein expression in breast cancer cells and breast tumor stroma is prognostic of poor disease outcome. Breast Cancer Res Treat. (2017) 166:85–94. 10.1007/s10549-017-4366-6 28717852

[44] Sui X Zhou H Zhu L Wang D Fan S Zhao W. CUL4A promotes proliferation and metastasis of colorectal cancer cells by regulating H3K4 trimethylation in epithelial-mesenchymal transition. Onco Targets Ther. (2017) 10:735–43. 10.2147/OTT.S118897 28223829 PMC5308582

[45] Wang Y Wen M Kwon Y Xu Y Liu Y Zhang P. CUL4A induces epithelial-mesenchymal transition and promotes cancer metastasis by regulating ZEB1 expression. Cancer Res. (2014) 74:520–31. 10.1158/0008-5472.CAN-13-2182 24305877 PMC3934357

[46] Kobayashi M Nagashio R Jiang SX Saito K Tsuchiya B Ryuge S. Calnexin is a novel sero-diagnostic marker for lung cancer. Lung Cancer. (2015) 90:342–5. 10.1016/j.lungcan.2015.08.015 26344721

[47] Patel VN Gokulrangan G Chowdhury SA Chen Y Sloan AE Koyutürk M. Network signatures of survival in glioblastoma multiforme. PLoS Comput Biol. (2013) 9:e1003237. 10.1371/journal.pcbi.1003237 24068912 PMC3777929

[48] Chen YC Hsiao CC Chen KD Hung YC Wu CY Lie CH. Peripheral immune cell gene expression changes in advanced non-small cell lung cancer patients treated with first line combination chemotherapy. PLoS ONE. (2013) 8:e57053. 10.1371/journal.pone.0057053 23451142 PMC3581559

[49] Li W Mao L Shu X Liu R Hao F Li J. Transcriptome analysis reveals differential immune related genes expression in bovine viral diarrhea virus-2 infected goat peripheral blood mononuclear cells (PBMCs). BMC Genomics. (2019) 20:516. 10.1186/s12864-019-5830-y 31226933 PMC6588900

