# Supplementary material for: Identification and Validation of Stage-Associated PBMC Biomarkers in Breast Cancer Using MS-Based Proteomics
Source: Front Oncol. 2020 Jul 24;10:1101. doi: 10.3389/fonc.2020.01101 (PMC7393188; doi:10.3389/fonc.2020.01101)
Supplement: Supplementary Figure 1 — The ROC analysis of CANX to detect the metastatic from non-metastatic stages of breast cancer patients. The AUC of CANX was 1 (p < 0.05). [file Data_Sheet_1.docx]

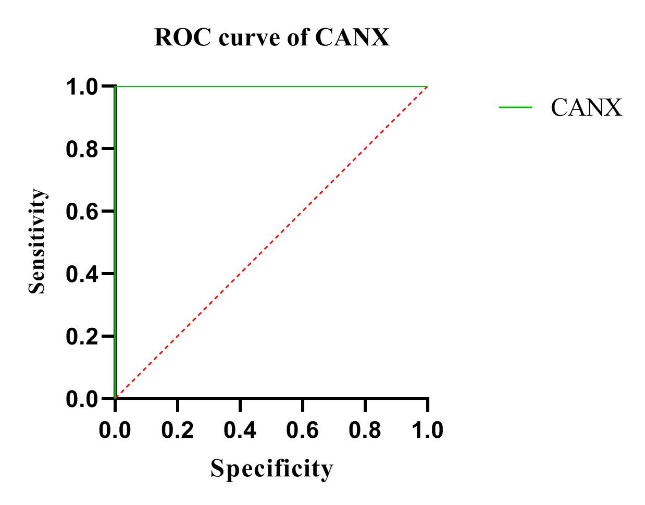


**Supplementary figure 1.** The ROC analysis of CANX to detect the metastatic from non-metastatic stages of breast cancer patients. The AUC of CANX was 1 (*p* < 0.05).
